# Chronic expanding hematoma of the left erector spinae muscle after stereotactic body radiotherapy for renal cell carcinoma: a case report

**DOI:** 10.1186/s13256-022-03612-3

**Published:** 2022-10-03

**Authors:** Yojiro Ishikawa, Takaya Yamamoto, Rei Umezawa, Noriyoshi Takahashi, Kazuya Takeda, Yu Suzuki, Keiichi Jingu

**Affiliations:** 1 Department of Radiation Oncology, Tohoku University Graduate School of Medicine, 1-1 Seiryo-chou, Aoba-ku, Sendai, Miyagi 980-8574 Japan; 2 Division of Radiology, Faculty of Medicine, Tohoku Medical and Pharmaceutical University, 1-15-1 Fukumuro, Miyagino-ku, Sendai, Miyagi 983-8536 Japan

**Keywords:** Chronic expanding hematomas, SBRT, Renal cell carcinoma

## Abstract

**Background:**

Hematomas that slowly increase in size for more than 1 month after the initial hemorrhage are referred to as chronic expanding hematomas. Chronic expanding hematoma can also occur after radiosurgery; however, there have been no reports about chronic expanding hematoma in the trunk after stereotactic body radiotherapy. We report a case of chronic expanding hematoma of the left erector spinae muscle after stereotactic body radiotherapy for renal cell carcinoma.

**Case presentation:**

A 74-year-old Japanese male complained of back pain 7 years after stereotactic body radiotherapy for renal cell carcinoma of the left kidney. There was no history of surgery or trauma to his back. After stereotactic body radiotherapy, there was no acute or late complication of more than grade 2. The renal cell carcinoma did not show shrinkage or progression, and he was diagnosed with stable disease on computed tomography. The patient remains in a stable disease condition 7 years after treatment without surgery or chemotherapy; however, he came to the hospital with gradually worsening back pain for several months. Computed tomography revealed the left erector spinae muscle to be swollen compared with the contralateral side at the third lumbar level. Ultrasonography showed a tumor of 30 mm in size without blood flow in the left paraspinal muscle. Positron emission tomography–computed tomography revealed uptake in the left paraspinal muscle. Pathological examination showed radiation-induced chronic expanding hematoma.

**Conclusions:**

We present the first case report of chronic expanding hematoma of the left erector spinae muscle after stereotactic body radiotherapy for renal cell carcinoma. Usually, stereotactic body radiotherapy for renal cell carcinoma would be considered unlikely to cause chronic expanding hematoma, but the introduction of dialysis and antiplatelet drugs may have increased the risk.

## Introduction

Hematoma that slowly increases in size for more than 1 month after the initial hemorrhage are referred to as chronic expanding hematoma (CEH). CEH is related to previous surgery or trauma [1], and can also occur after radiotherapy. There have been reports of CEH in the brain after treatment with a gamma knife [2, 3]; however, there have been no reports about CEH in the trunk after radiotherapy. It appears that radiotherapy can induce some changes leading to CEH, but this remains an uncommon finding.

We report a case of CEH of the left erector spinae muscle after stereotactic body radiotherapy (SBRT) for renal cell carcinoma (RCC). This article was previously uploaded as a preprint on Research Square [4].

## Case report

A 74-year-old Japanese male complained of back pain. There was no history of drinking or smoking. The patient had a medical history of dialysis due to nephrotic syndrome, operation of abdominal aortic aneurysm, coronary stenting, pacemaker implantation, and RCC. The patient did not have a history of medical or surgical therapy for tuberculosis. He was taking aspirin and clopidogrel sulfate, and his family history included duodenal cancer in his father.

Seven years ago, the patient was diagnosed with RCC (cT1aN0M0, cStage I). His renal function had already failed, and the patient was put on dialysis. The surgeons did not recommend resection of the right kidney owing to concerns about several complications, despite the fact that the patient had an Eastern Cooperative Oncology Group Performance Score of 0. The surgeons recommended ablation of the tumor; however, the patient refused to undergo ablation due to concerns about the treatment side effects. The fact that this was before the introduction of ablation therapy for renal cancer at our institution also impacted this patient.

**Fig. 1:**
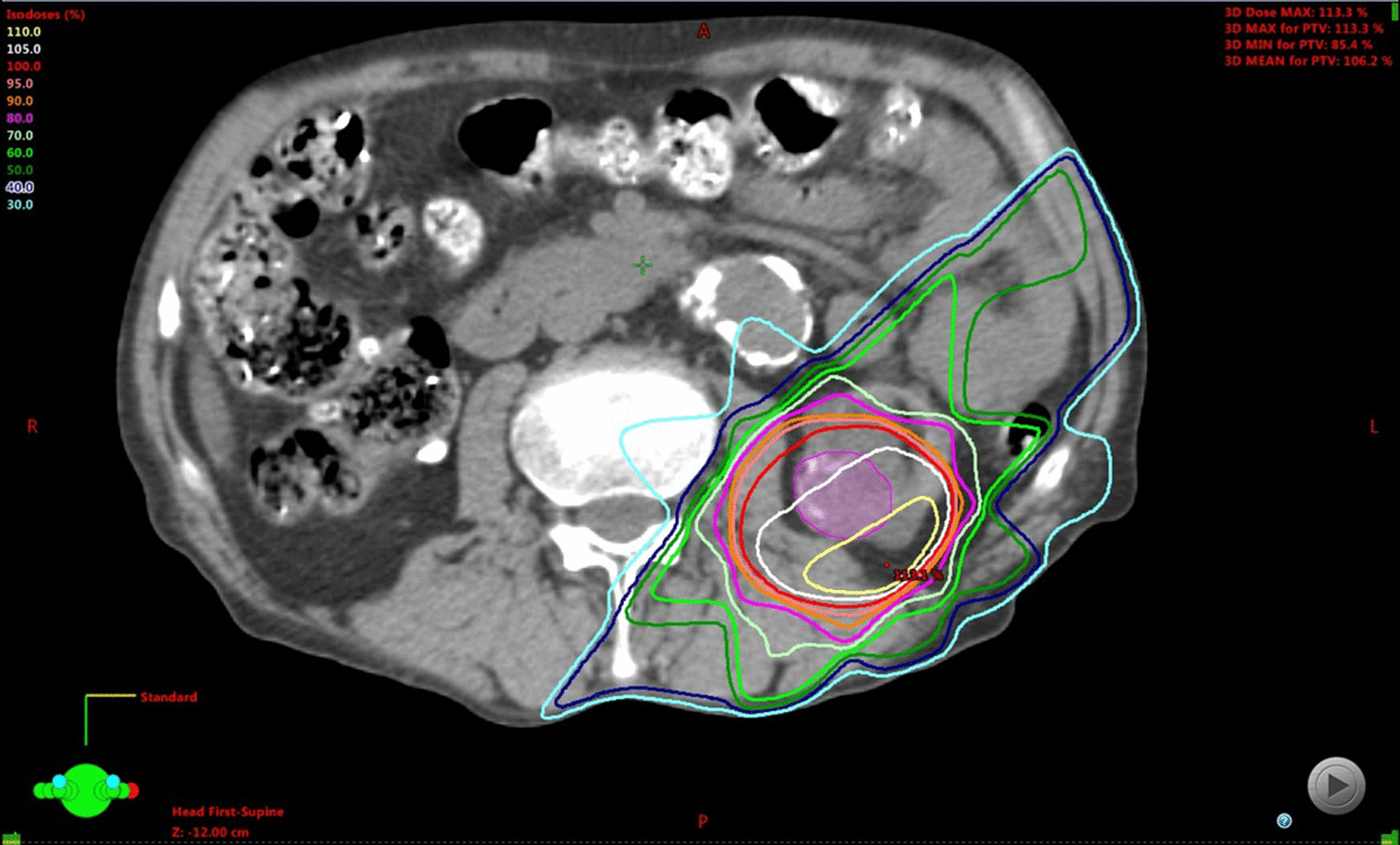
Axial image of dose distribution of stereotactic body radiotherapy (SBRT). Stereotactic body radiotherapy was performed with seven non-coplanar static 6 MV X-ray beams to the lesion of the right kidney using daily cone-beam computed tomography scans before each fraction. The patient was prescribed 70 Gy/10 fractions for the isocenter.

Therefore, SBRT was recommended for the right kidney tumor, which the patient accepted. We performed SBRT, which was created with a three-dimensional radiotherapy planning system (Eclipse, Varian Medical Systems, Palo Alto, CA). Respiratory tumor movement was measured using continuous X-ray images in a simulator. Gross tumor volume (GTV) was defined as the visible tumor on the planning computed tomography (CT) image. The CTV was defined as the GTV plus 3 mm because patients only underwent non-contrast-enhanced CT. The planning target volume (PTV) margin of 5 mm was added to CTV. Prescription doses were based on a previous report [5]: 70 Gy in 10 fractions covering 95% of the PTV (D95) was delivered using 6 MV X-rays. Dose constraints for organs at risk conformed to previous reports [6] (Fig. 1). An acute side effect of grade 1 dermatitis occurred after SBRT, but there was no acute or late complication of more than grade 2. The RCC did not show shrinkage or progression and the patient was diagnosed with stable disease on CT. The patient remains stable 7 years after treatment without surgery or chemotherapy; however, the patient came to the hospital with gradually worsening back pain for several months.

**Fig. 2:**
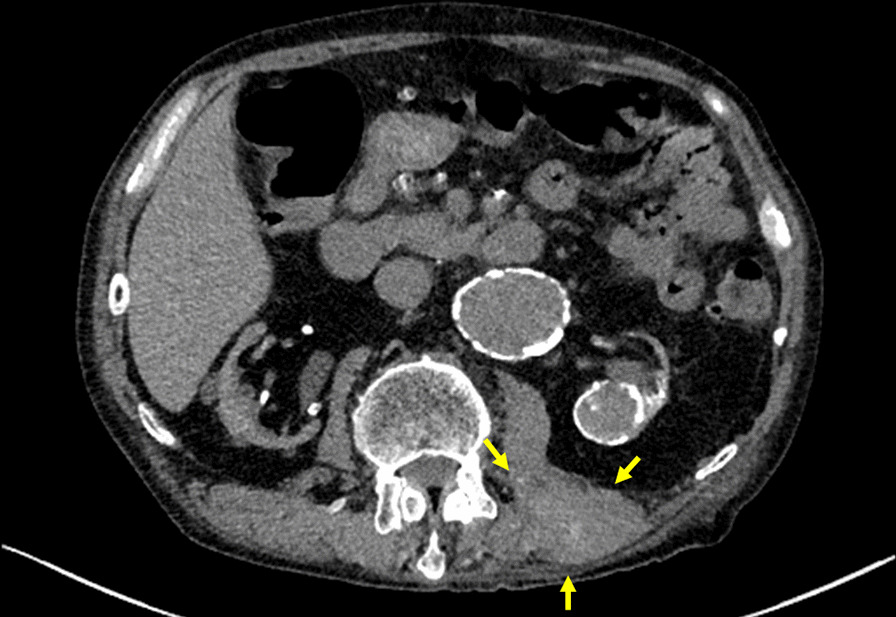
Axial enhanced computed tomography scan images showed a tumor that had spread to the left erector spinae muscle. The lesion appeared to have a nodular structure including high and low attenuation areas (yellow arrow).

**Fig. 3:**
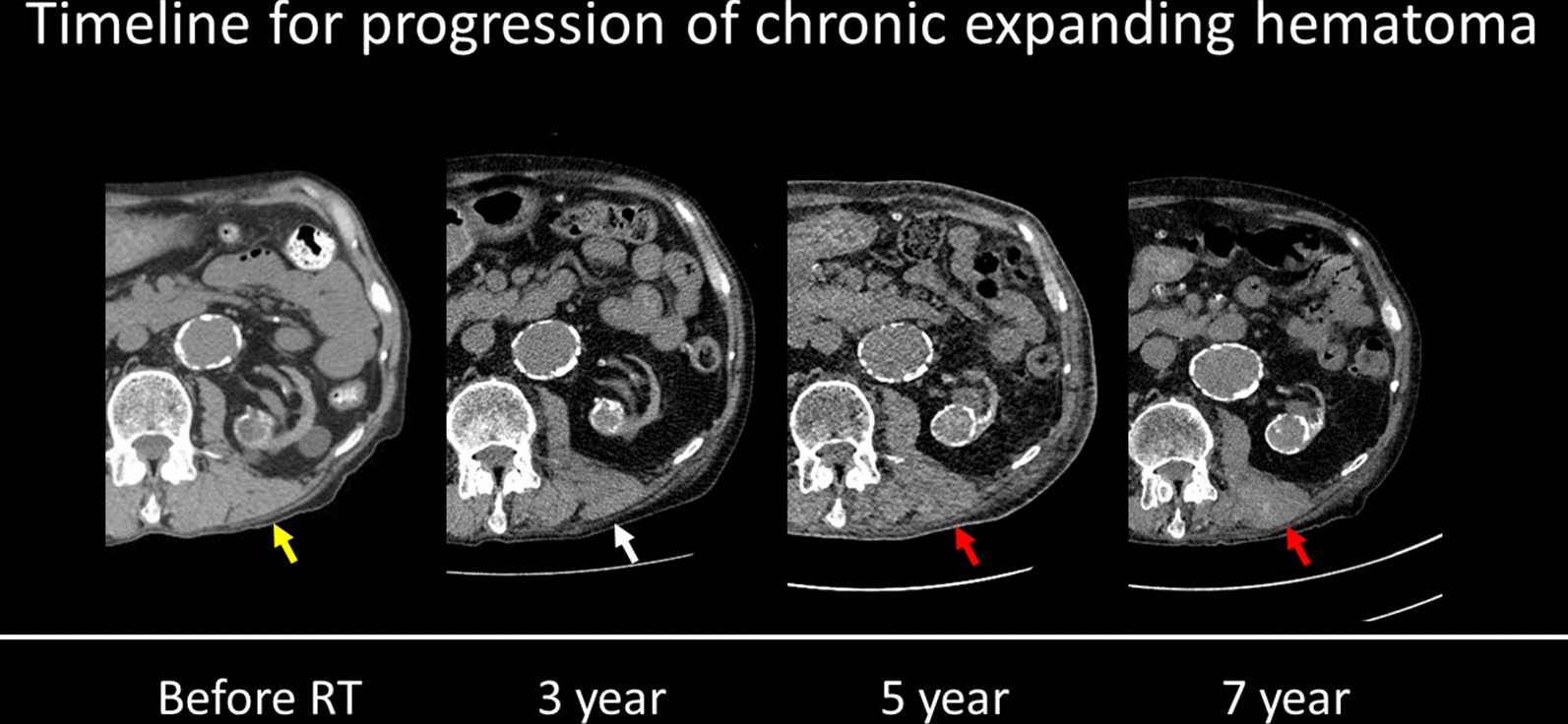
Timeline for progression of the chronic expanding hematoma on computed tomography (yellow arrow). Before SBRT, the left erector spinae muscle was normal. The muscle gradually became atrophic 3 years after SBRT (white arrow). Finally, the muscle was swollen between 5 and 7 years after SBRT (red arrow).

**Fig. 4:**
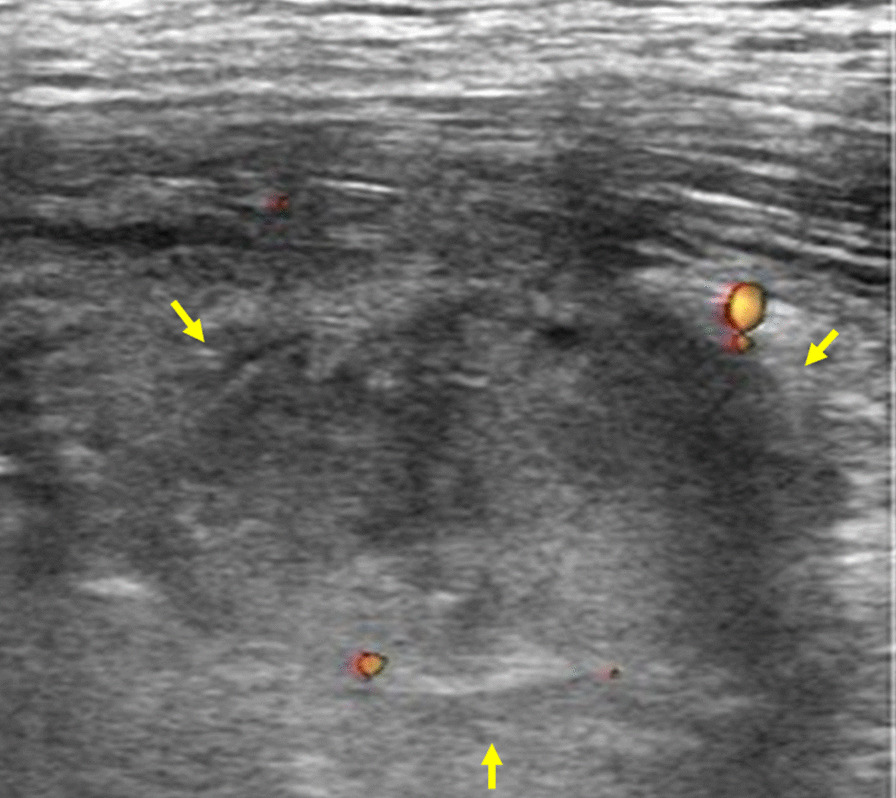
Ultrasonography of left erector spinae muscle showed an iso-hypoechoic tumor 30 mm in size without blood flow (yellow arrow).

**Fig. 5:**
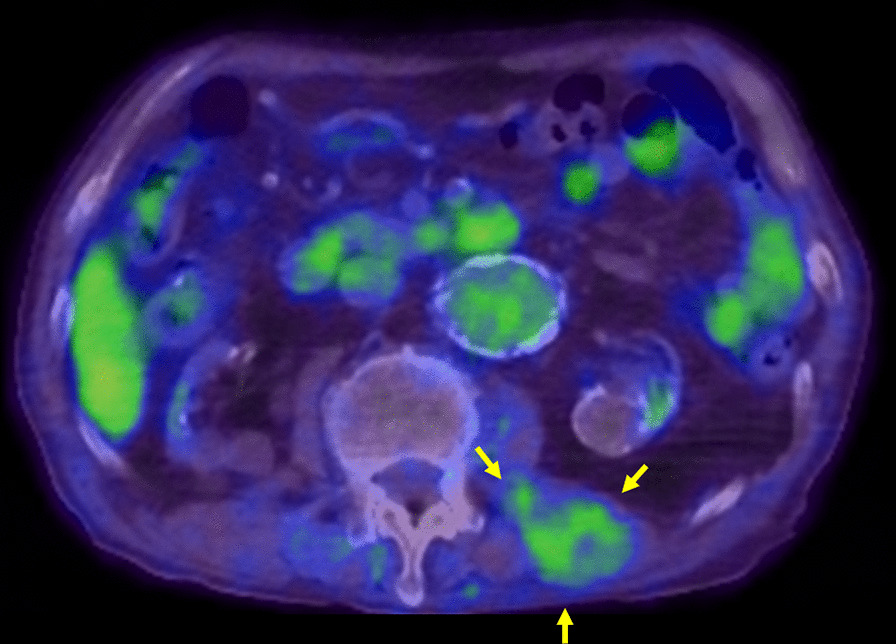
Positron emission tomography–computed tomography showed uptake of 18F-2-fluoro-2-deoxy-D-glucose in the left erector spinae muscle with a maximum standardized uptake value (SUVmax) of 2.8 (yellow arrow).

CT revealed that the left erector spinae muscle was swollen compared with the contralateral side at the third lumbar level (Fig. 2). The lesion appeared to have a nodular structure including high and low attenuation areas. Retrospectively, it had been clarified 5 years ago, but the change was gradual (Fig. 3). Ultrasonography showed a tumor 30 mm in size without blood flow in the left paraspinal muscle (Fig. 4). Magnetic resonance imaging was not performed because he was a pacemaker patient. Positron emission tomography–CT (PET–CT) revealed uptake of 18F-2-fluoro-2-deoxy-D-glucose (FDG) in the left paraspinal muscle (maximum standardized uptake value of 2.8) (Fig. 5). We therefore suspected a malignant tumor, a benign tumor such as schwannoma, vascular malformations, or chronic expanding hematoma.

Pathological examination of biopsy specimens from the lesion of the left erector spinae muscle showed bleeding and fibrine precipitation. In addition, atrophy and glassy fibrosis of the striated muscle due to radiotherapy were observed. The clinical diagnosis was radiation-induced CEH.

We performed conservative therapy with medication alone because the tumor had been gradually growing. One year after starting observation, the patient died due to subdural hemorrhage after a fall and progression of renal failure.

## Discussion

CEH is related to a history of surgery or trauma. In addition, CEH in the chest occurs in patients with a history of treatment for tuberculosis [7, 8]. It is not uncommon for CEH to occur after surgery, trauma, or tuberculosis; however, no case similar to this was found in a PubMed search of studies in English (available at http://www.ncbi.nlm.nih.gov/pubmed/) using “chronic expanding hematoma” with “stereotactic body radiotherapy” as index words. This is the first report of CEH of the left erector spinae muscle after SBRT for RCC.

The durations between previous episodes and the first diagnosis of CEH can vary from 1 month to decades [9]. CEH can result from the stimulation of blood and its degradation products, leading to repeated exudation or bleeding from the capillaries of granulation tissue. The factors that trigger this behavior in some hematomas are unclear [10]. Late complications after intracranial radiotherapy (RT) occur in 1–5% of patients [11, 12]. They include delayed cysticercosis, CEH, and cavernous hemangioma; however, the mechanism is not clear [13–16]. Extracranial RT can cause radiation-induced late bleeding or pseudoaneurysm [4, 17, 18]. Anticoagulant therapy has been shown to be associated with the incidence of CEH. Treatment of CEH is total resection, however, this is reportedly difficult due to the presence of fibrous adhesions [19].

In a PubMed search of English literature, 109 reports had “chronic expanding hematoma” in the title. Of these, 73 were case reports on CEH and 15 were case studies. Of the 133 cases found in the present review (Table 1), 94 (71%) of the cases were males and 39 (29%) were females. The median age of the patients was 65 years (range 8–89 years). The reported clinical latent periods ranged widely from 1 to 660 months (median of 252 months). The most common lesion site was the thorax including the chest wall in 59 (44.4%) of the patients. Two (1.5%) and 36 (27.0%) patients had lesions in the upper and lower extremities, respectively, and 13 (9.7%) and 16 (12.0%) patients had lesions in the abdomen and pelvis, respectively. Forty (30.0%) and 46 (36.8%) patients had antecedent episodes of trauma and surgery, respectively. The most common therapy for CEH was total resection; however, six (4.5%) patients did not undergo resection. In six (4.5%) patients, CEH was the cause of death. The present case was on antiplatelet medication, which may have increased the risk of bleeding. Although few papers explicitly point this out, seven (5.2%) patients had undergone anticoagulant therapy or antiplatelets, and five (3.8%) patients were on dialysis.

**Table 1:** Review of the 133 cases with chronic expanding hematoma

	*N*=133
**Sex**	
Male	94 (71%)
Female	39 (29%)
**Age (years)**	
Median	65 (48–89)
**Location**	
Upper extremities	2 (1.5%)
Lower extremities	36 (27.1%)
Thorax	59 (44.4%)
Abdomen	13 (9.7%)
Pelvis	16 (12.0%)
Head and neck	3 (2.3%)
Other	4 (3.0%)
**Antecedent episode**	
Trauma	40 (30.0%)
Surgery	49 (36.8%)
Tuberculosis	32 (24.1%)
Radiation therapy	3 (2.3%)
Other	2 (1.5%)
**Range from antecedent episode (months)**	
Median	252 (1–660)
**Treatment**	
Surgery	115 (86.4%)
Observation	6 (4.5%)
Embolization	15 (11.3%)
Unknown	11 (8.27%)

Our review found that three cases (2.2%) had a history of radiotherapy after surgery. In two cases with a history of radiotherapy, the irradiation area and dose were not clear. Sakamoto *et al.* reported that CEH occurred in the foot after an operation with adjuvant radiotherapy (84 Gy) [20]. There have been reports of CEH in the brain after treatment with gamma knife for cerebral arteriovenous malformations [2, 3]. However, this is the first report of CEH after SBRT.

Hemodialysis (HD) patients are at an increased risk of bleeding because of uremic bleeding tendency and systemic anticoagulation caused by intermittent heparinization [21]. Therefore, HD might be associated with the incidence of CEH. CEH occurred in some patients undergoing HD. Our patient had a medical history of HD due to nephrotic syndrome.

Because this was a case study, it is difficult to definitively conclude the CEH was due to SBRT. However, it is possible that CEH occurs in some patients after SBRT. Since SBRT has recently been increasing, SBRT-induced CEH is considered an important complication.

## Conclusions

We report a case of CEH of the left erector spinae muscle after SBRT for RCC. CEH can result from the stimulation of blood and its degradation products, leading to repeated exudation or bleeding from the capillaries of granulation tissue. Usually, SBRT for RCC would be considered unlikely to cause CEH, but the introduction of dialysis and antiplatelet drugs may have increased the risk of CEH. CEH often occurs over a very long course, and requires careful clinical follow-up based on the irradiated field of SBRT for RCC.

## Data Availability

The data include individual patient data, but the data are available from the corresponding authors upon reasonable request.
